# A Treatise on Diseases of the Eye

**Published:** 1874-02

**Authors:** 


*A Treatise on the Diseases of The Eye* By J. Soelberg Wells, F. R. C. S., etc. Second American, from the Third English Edition. Philadelphia: Henry C. Lea, 1874. Buffalo: T. Butler & Son.

The work of Mr Wells is so well known that it is unnecessary for us to give an extended notice of it at this time. The present edition embraces several additions to the former and will be found to be fully up to the present standard of ophthalmological science. The work has passed through the press under the supervision of Dr. J. Minnis Hays, who has made a few additions upon points which have escaped the notice of the author. As a complete treatise upon Diseases of the Eye, the work of Mr. Wells has no superior in the English language, and we bid a hearty welcome to the second edition of a work which has deservedly gained the admiration and favor of all students of Ophthalmology.

